# Expanded use of triclosan-coated sutures for surgical site infection prevention in oesophageal cancer surgery

**DOI:** 10.1186/s12893-026-03723-4

**Published:** 2026-04-20

**Authors:** Kohei Fujita, Ryo Ogawa, Shohei Hayashi, Eri Tsuji, Sunao Ito, Shunsuke Hayakawa, Reo Sato, Yushi Yamakawa, Takafumi Sato, Hiroyuki Sagawa, Shuji Takiguchi

**Affiliations:** Department of Gastroenterological Surgery, Nagoya City University Graduate School of Medical Sciences and Medical School, 1 Kawasumi, Mizuhocho, Mizuhoku, Nagoya, Aichi 467-8601 Japan

**Keywords:** Oesophageal cancer, Surgical site infection, Triclosan-coated sutures, Antimicrobial sutures

## Abstract

**Background:**

Surgical site infection (SSI) remains one of the most common and serious complications following oesophageal cancer surgery, contributing to prolonged hospitalisation, increased healthcare costs, and reduced postoperative quality of life. Although triclosan-coated antimicrobial sutures have been shown to reduce SSIs in various surgical fields by inhibiting bacterial adhesion and biofilm formation, their use in oesophageal cancer surgery has not been adequately evaluated. This study aimed to assess the efficacy of triclosan-coated sutures in preventing SSIs when applied broadly during oesophageal cancer surgery.

**Methods:**

This retrospective cohort study enrolled 182 patients who underwent oesophageal cancer surgery between January 2021 and December 2024. Patients were divided into the conventional (*n* = 135) and triclosan-coated sutures groups (*n* = 47). In the triclosan group, antimicrobial sutures were used for skin and fascial closure, as well as in intra-abdominal applications—reinforcement of the gastric conduit staple line and anastomoses. SSI incidence was classified per the Centres for Disease Control and Prevention guidelines. Risk factors were identified using univariate and multivariate logistic regression analyses. Microbiological cultures of the infected sites were analysed.

**Results:**

The overall SSI rate was significantly lower in the triclosan group than that in the conventional group (10.6% vs. 25.9%, *P* = 0.0211). The incidence of superficial, deep, and organ/space infections was lower in the triclosan group than that in the conventional group, albeit without statistical significance. In this exploratory multivariable logistic regression analysis, conventional suture use remained statistically associated with SSI [odds ratio (OR) 3.09, 95% confidence interval (CI) 1.08–8.82, *P* = 0.0350].

**Conclusions:**

Triclosan-coated sutures were associated with a reduced incidence of SSIs after oesophageal cancer surgery. Given the retrospective design and the limited number of events, these findings should be interpreted cautiously. Further prospective multicentre studies are warranted to validate these results.

**Supplementary Information:**

The online version contains supplementary material available at 10.1186/s12893-026-03723-4.

## Background

Oesophageal cancer is among the leading causes of cancer-related mortality, ranking sixth globally, and accounting for ~ 6% of all cancer-related deaths [1]. Recent advances in surgical techniques and perioperative management have improved the short-term outcomes of surgical interventions for oesophageal cancer, with a reported mortality rate of ~ 3.5% [2, 3]. Surgical site infection (SSI) is the most common postoperative complication of oesophageal cancer surgery, affecting ~ 22.1% of patients and imposing significant physical, psychological, and social burdens on patients and healthcare systems, thus highlighting the need for effective preventive strategies [4].

Triclosan (polychlorophenoxyphenol) is an antimicrobial agent with a well-established safety profile, which is widely used as a preservative in products such as toothpaste and soaps. Triclosan-coated sutures are primarily used for skin and fascial closure and can reportedly reduce the risk of SSIs by inhibiting bacterial adhesion and biofilm formation. Several randomised controlled trials and meta-analyses have demonstrated that the use of triclosan-coated sutures, such as Vicryl^®^ Plus and PDS^®^ Plus (Ethicon, Johnson & Johnson, Tokyo, Japan), for fascial and dermal closure significantly decreased SSI rates across various surgical fields [5–9]. The 2017 Centres for Disease Control and Prevention (CDC) guidelines for the prevention of SSIs recommended the use of triclosan-coated sutures for fascial and skin closure as part of a comprehensive risk reduction strategy [10]. However, to date, no studies have evaluated the efficacy of antimicrobial sutures in oesophageal cancer surgery.

Oesophagectomy is a highly invasive procedure involving multiple anatomical regions, including the cervical, thoracic, and abdominal regions, inherently increasing the risk of complications such as SSIs [3, 4]. Risk factors for SSIs during oesophageal surgery include hypoalbuminemia, diabetes mellitus, prolonged operative time, and smoking [4, 11, 12]. The surgical complexity of oesophagectomy is challenging for SSI prevention while simultaneously presenting an opportunity for targeted intraoperative interventions, including the expanded use of triclosan-coated sutures beyond superficial closure to include deeper surgical sites such as anastomoses and surrounding organs. Although these sutures are effective against certain Gram-positive and -negative organisms, they may not sufficiently suppress bacteria with low susceptibility, such as anaerobes and Enterococcus species [13, 14], potentially increasing the incidence of culture-negative infections or altering the microbial spectrum. Thus, active microbiological surveillance is essential for monitoring these trends and guiding perioperative antimicrobial strategies. Here, we evaluated whether the use of triclosan-coated sutures for superficial closure as well as in deeper surgical sites, including anastomoses and surrounding organs, could reduce the incidence of SSIs in patients undergoing oesophageal cancer surgery.

## Methods

### Patients

This retrospective cohort study included all patients who underwent oesophageal cancer surgery at Nagoya City University Hospital (Nagoya, Japan) between January 2021 and December 2024. In total, 182 consecutive patients were enrolled.

### Operative procedures

At our institution, thoracic and abdominal oesophageal cancers are primarily treated using minimally invasive McKeown oesophagectomy. The thoracic phase involved thoracoscopic oesophagectomy and mediastinal lymph node dissection. In the abdominal phase, laparoscopic lymphadenectomy was performed, and the oesophagus was mobilised and exteriorised through the abdominal incision. A gastric conduit was created extracorporeally using a linear stapler, and the staple line was reinforced with approximately 30 interrupted seromuscular sutures. Subsequently, the retrosternal route was dissected, and the gastric conduit was delivered to the cervical region. In the cervical phase, reconstruction was performed using either the gastric conduit or a segment of the jejunum, depending on individual cases. Anastomoses were performed either by stapling or hand sewing, with additional reinforcement sutures, even in stapled cases. For linear-stapled anastomoses (modified Collard technique), reinforcement sutures were placed at sites with overlapping staple lines. For circular-stapled anastomoses, additional reinforcement sutures were placed in areas deemed to have thin walls by the surgeon. The cervical wound was closed in two layers: the platysma and skin. A collar incision was made for cervical oesophageal cancer, and lymphadenectomy was performed, followed by transection of the cervical oesophagus. A vascularised, free jejunal graft was harvested from the abdomen for use in reconstruction. The anastomotic technique (hand-sewn, stapled, or cervical oesophagostomy) was selected at the surgeon’s discretion.

Prior to January 2024, triclosan-coated sutures (Vicryl^®^ Plus or PDS^®^ Plus, Ethicon, Johnson & Johnson, Tokyo, Japan) were not used at our institution. Fascial closure was performed using interrupted sutures with conventional Vicryl^®^, and the skin was closed with subcuticular sutures using conventional PDS^®^. Before skin closure, the anastomotic site and subcutaneous tissues were irrigated with sterile saline in all cases. This procedure was standardised throughout the study period and performed according to routine institutional protocol to prevent superficial SSIs. Since January 2024, triclosan-coated sutures such as Vicryl^®^ Plus or PDS^®^ Plus have been used for all suturing procedures, including skin and fascial closure, seromuscular reinforcement of the gastric conduit staple line, hand-sewn anastomoses, and reinforcement sutures around stapled anastomoses.

### Surgical site infection prevention bundle

A standardised SSI prevention bundle, based on the National Institute for Health and Care Excellence (NICE) guidelines, was consistently applied throughout the study period [15]. The core components included preoperative showering or bathing, use of alcohol-based chlorhexidine for skin antisepsis, timely prophylactic antibiotic administration (typically cefazolin 30–60 min before skin incision, with intraoperative redosing as indicated), maintenance of normothermia, and postoperative wound care education. The complete details are listed in Additional file 1.

### Outcomes and statistical analysis

The primary outcome of this study was the incidence of SSI, categorised into superficial, deep, and organ/space types according to CDC definitions [10, 16]. SSI assessment was performed daily during postoperative ward rounds by the attending surgeons. When SSI was suspected, findings were documented in the medical records, and wound cultures were obtained for microbiological evaluation. The diagnostic criteria are detailed in Additional file 2. To evaluate factors associated with SSI development, univariate and multivariate logistic regression analyses were performed to examine patient background variables, surgical factors, and the use or non-use of triclosan-coated sutures. Wound specimens were obtained at the time of SSI diagnosis and were subjected to aerobic and anaerobic bacterial cultures using standard microbiological techniques. The effect of triclosan-coated sutures on the distribution of causative organisms was also assessed. Statistical analyses were performed using JMP software (v. 13.2.1; SAS Institute, Cary, NC, USA). Associations between individual clinical variables and SSI occurrence were evaluated using the chi-squared test or Student’s t-test, as appropriate. Fisher’s exact test was applied when expected cell counts were small. Variables found to be significant in the univariate analysis were further examined using multivariate nominal logistic regression. Fifteen variables were evaluated in the univariate analysis: suture type (conventional vs. triclosan-coated), sex, age (≥ 75 years), body mass index (BMI) ≥ 25 kg/m^2^, hypertension, diabetes mellitus, respiratory disease, atherosclerotic cardiovascular disease, history of cervical or thoracic surgery, smoking history, American Society of Anaesthesiologists’ (ASA) physical status (ASAPS) (≥ 3), serum albumin level (< 3.0 g/dL), receipt of neoadjuvant therapy, operative time (≥ 600 min), and use of minimally invasive surgery (MIS). Variables with a P-value < 0.1 in the univariate analysis—including suture type, sex, age, diabetes mellitus, smoking history, and ASA-PS classification—were subsequently included in the multivariate logistic regression model.

## Results

### Clinicopathological characteristics

A total of 182 patients were included in this study: 135 underwent surgery using conventional sutures between January 2021 and December 2023, and 47 were treated with triclosan-coated sutures between January and December 2024. The baseline characteristics of each group are presented in Table 1. No significant differences were observed between the two groups in terms of age (70.0 ± 10.2 vs. 70.0 ± 7.7 years, *P* = 0.8730), sex (male: 77.8% vs. 80.1%), or BMI: 20.6 ± 3.25 vs. 20.8 ± 3.37 kg/m^2^. Comorbidities, including diabetes mellitus, respiratory disease, atherosclerotic cardiovascular disease, smoking history, previous cervical or thoracic surgery, and tumour location and stage (TNM classification), were similarly distributed between the groups. However, the prevalence of hypertension was significantly higher in the conventional sutures group (45.2% vs. 25.5%, *P* = 0.0157). In contrast, a greater proportion of patients in the triclosan group had an ASA-PS classification of grade 1 (55.3% vs. 34.8%, *P* = 0.0342). Although the rate of preoperative treatment tended to be higher in the triclosan group, the difference was not statistically significant (*P* = 0.0753).

**Table 1:** Preoperative characteristics of patients undergoing oesophageal cancer surgery

	Conventional sutures (*n* = 135)	Triclosan-coated sutures (*n* = 47)	*P*-value
Age, years, mean	70.0 ± 10.2	70.0 ± 7.7	0.8730
Sex			0.6554
Male	105 (77.8%)	38 (80.1%)	
Female	30 (22.2%)	9 (19.1%)	
BMI	20.6 ± 3.25	20.8 ± 3.37	0.8392
Pre-existing medical condition			
Diabetes mellitus	13 (9.6%)	4 (8.5%)	0.8188
Hypertension	61 (45.2%)	12 (25.5%)	0.0157
Respiratory diseases	9 (6.7%)	3 (6.4%)	0.9460
Atherosclerotic cardiovascular disease	15 (11.1%)	6 (8.6%)	0.75
Smoking history	110 (81.5%)	41 (87.2%)	0.3543
Preoperative treatment	72 (53.3%)	32 (68.1%)	0.0753
History of cervical and thoracic surgery	9 (6.7%)	5 (10.6%)	0.3940
ASA score			0.0342
1	47 (34.8%)	26 (55.3%)	
2	81 (60.0%)	18 (38.3%)	
3	7 (5.2%)	3 (6.4%)	
Lesion location			0.8239
Cervical	11 (8.1%)	4 (8.5%)	
Thoracic	118 (87.4%)	42 (89.4%)	
Abdominal	5 (3.7%)	1 (2.1%)	
TNM stage			0.4810
0	5 (3.7%)	4 (8.5%)	
1	36 (26.7%)	13 (27.7%)	
2	31 (23.0%)	14 (29.8%)	
3	54 (40.0%)	14 (29.8%)	
4	9 (6.7%)	2 (4.3%)	

Staging was performed according to the 8th edition of the Union for International Cancer Control TNM classification

*ASA* American Society of Anaesthesiologists, *BMI* body mass index

### Operative characteristics and postoperative complications

The operative details and postoperative complications are summarised in Table 2. Operative time (567 ± 145 min vs. 595 ± 126 min, *P* = 0.2525), estimated blood loss (203 ± 262 mL vs. 186 ± 222 mL, *P* = 0.6989), and rate of MIS (93.3% vs. 89.4%, *P* = 0.3940) did not differ significantly between the conventional and triclosan groups. The choice of anastomotic technique was similar between the groups. The incidence of severe complications (Clavien–Dindo grade ≥ 3) was 21.5% (29 cases) in the conventional group and 17.0% (8 cases) in the triclosan group, not statistically significant (*P* = 0.6571). Among the non-SSI complications, the rates of sputum retention requiring intervention and pneumonia were 4.4% (6 cases) and 2.2% (3 cases), respectively, in the conventional group and 0% and 0%, respectively, in the triclosan group. Other complications included pneumothorax (4.3%), chylothorax, and recurrent laryngeal nerve palsy, which were observed only in the triclosan group; however, their incidence was low, and no notable differences in the overall complication trends were found between the two groups. Postoperative hospitalisation was also comparable between the two groups (26.4 ± 14.0 days vs. 25.8 ± 19.8 days, *P* = 0.8313).

**Table 2:** Operative characteristics and postoperative complications

	Conventional sutures (*n* = 135)	Triclosan-coated sutures (*n* = 47)	*P*-value
Operative time, min, mean ± SD	567 ± 145	595 ± 126	0.2525
Estimated blood loss, mL, mean ± SD	203 ± 262	186 ± 222	0.6989
Approach			0.3940
Minimally invasive oesophagectomy	126 (93.3%)	42 (89.4%)	
Open oesophagectomy	9 (6.7%)	5 (10.6%)	
Anastomosis techniques			0.8141
Circular stapled anastomosis	30 (22.2%)	11 (23.4%)	
Linear stapled anastomosis	87 (64.4%)	31 (66.0%)	
Hand-sewn anastomosis	11 (8.1%)	2 (4.3%)	
No anastomosis	7 (5.2%)	3 (6.4%)	
Complications (CD ≥ 3)	29 (21.5%)	8 (17.0%)	0.5067
Leakage	20 (14.8%)	3 (6.4%)	0.1110
Anastomotic stricture	0 (0%)	1 (2.1%)	0.0989
Impaired sputum clearance	6 (4.4%)	0 (0%)	0.0559
Pneumonia	3 (2.2%)	0 (0%)	0.1785
Empyema	0 (0%)	1 (2.1%)	0.0989
Pneumothorax	0 (0%)	2 (4.3%)	0.0192
Chylothorax	1 (0.7%)	0 (0%)	0.4388
Pancreatic fistula	1 (0.7%)	0 (0%)	0.4388
Recurrent laryngeal nerve palsy	2 (1.5%)	1 (2.1%)	0.7706
Postoperative duration of hospitalisation (days)	26.4 ± 14.0	25.8 ± 19.8	0.8313

Complications were graded according to the Clavien–Dindo (CD) classification

*SSI* surgical site infection

### Incidence of surgical site infection

The incidence of SSIs within 30 days postoperatively is shown in Table 3. In all three categories, the incidence of SSI was lower in the triclosan group than that in the conventional group, although none of the individual differences was significant (superficial: 7.4% vs. 4.3%, *P* = 0.4335; deep: 2.2% vs. 0%, *P* = 0.1785; organ/space: 16.3% vs. 6.4%, *P* = 0.0688). In contrast, the overall incidence of SSI (total SSI) was significantly lower in the triclosan group than that in the conventional group (10.6% vs. 25.9%, *P* = 0.0211).

**Table 3:** Incidence of surgical site infection and multivariate logistic regression analysis of risk factors

	Conventional sutures (*n* = 135)	Triclosan-coated sutures (*n* = 47)	*P*-value
Superficial incisional SSI	10 (7.4%)	2 (4.3%)	0.7338
Deep incisional SSI	3 (2.2%)	0 (0%)	0.5698
Organ/space SSI	22 (16.3%)	3 (6.4%)	0.0688
Total SSI	35 (25.9%)	5 (10.6%)	0.0211
Leakage	20 (14.8%)	3 (6.4%)	0.1110
Circular stapled anastomosis	6 (20.0%)	0 (0%)	0.1672
Linear stapled anastomosis	11 (12.6%)	2 (6.5%)	0.5095
Hand-sewn anastomosis	3 (27.3%)	1 (50%)	1.0000

The incidence of anastomotic leakage (considered organ/space SSI according to the CDC criteria) was 14.8% (20 cases) in the conventional group and 6.4% (3 cases) in the triclosan group, with no significant difference between them. The incidence of anastomotic leakage did not differ significantly among the different anastomotic techniques

### Univariate and multivariate analyses

To identify the risk factors for SSI, univariate and multivariate logistic regression analyses were performed using a nominal logistic model. Univariate analysis revealed that the use of conventional sutures [odds ratio (OR) 2.94, *P* = 0.0353], male sex (OR 4.19, *P* = 0.0231), and age ≥ 75 years (OR 2.09, *P* = 0.0435) were significantly associated with SSI occurrence. In this exploratory multivariable logistic regression analysis, the use of conventional sutures remained statistically associated with SSI [OR 3.09, 95% confidence interval (CI) 1.08–8.82, *P* = 0.0350]. The results of the univariate and multivariate analyses are detailed in Table 4.

**Table 4:** Univariate and multivariate analyses of surgical site infection

	Univariate			Multivariate		
	OR	95% CI	*P*-value	OR	95% CI	*P*-value
Type of sutures (Conventional sutures)	2.94	1.08–8.02	0.0353	3.09	1.08–8.82	0.0350
Sex (male)	4.19	1.22–14.4	0.0231	3.17	0.89–11.6	0.0806
Age (≥ 75 years)	2.09	1.02–4.26	0.0435	1.95	0.90–4.21	0.0892
BMI (≥ 25 kg/m^2^)	1.75	0.62–4.95	0.2903			
Hypertension	1.47	0.73–2.99	0.2818			
Diabetes mellitus	2.80	0.99–7.90	0.0520	2.30	0.76–7.02	0.1410
Respiratory disease	0.31	0.38–2.44	0.2632			
Atherosclerotic cardiovascular disease	1.94	0.72–5.19	0.1874			
History of cervical or thoracic surgery	1.47	0.43–4.95	0.5373			
History of smoking	3.03	0.87–10.5	0.0815	2.65	0.70–10.1	0.1516
ASA (≥ 2)	2.04	0.95–4.41	0.0687	1.33	0.58–3.07	0.5015
Alb (< 3.0)	0.61	0.20–1.91	0.3983			
Preoperative treatment	0.89	0.44–1.81	0.7566			
Operative time (≥ 600 min)	1.71	0.84–3.49	0.1426			
Minimally Invasive Surgery	3.93	0.50–30.1	0.1940			

*BMI* body mass index, *Alb* albumin, *ASA* American Society of Anaesthesiologists

### Causative organisms in SSI

**Fig. 1:**
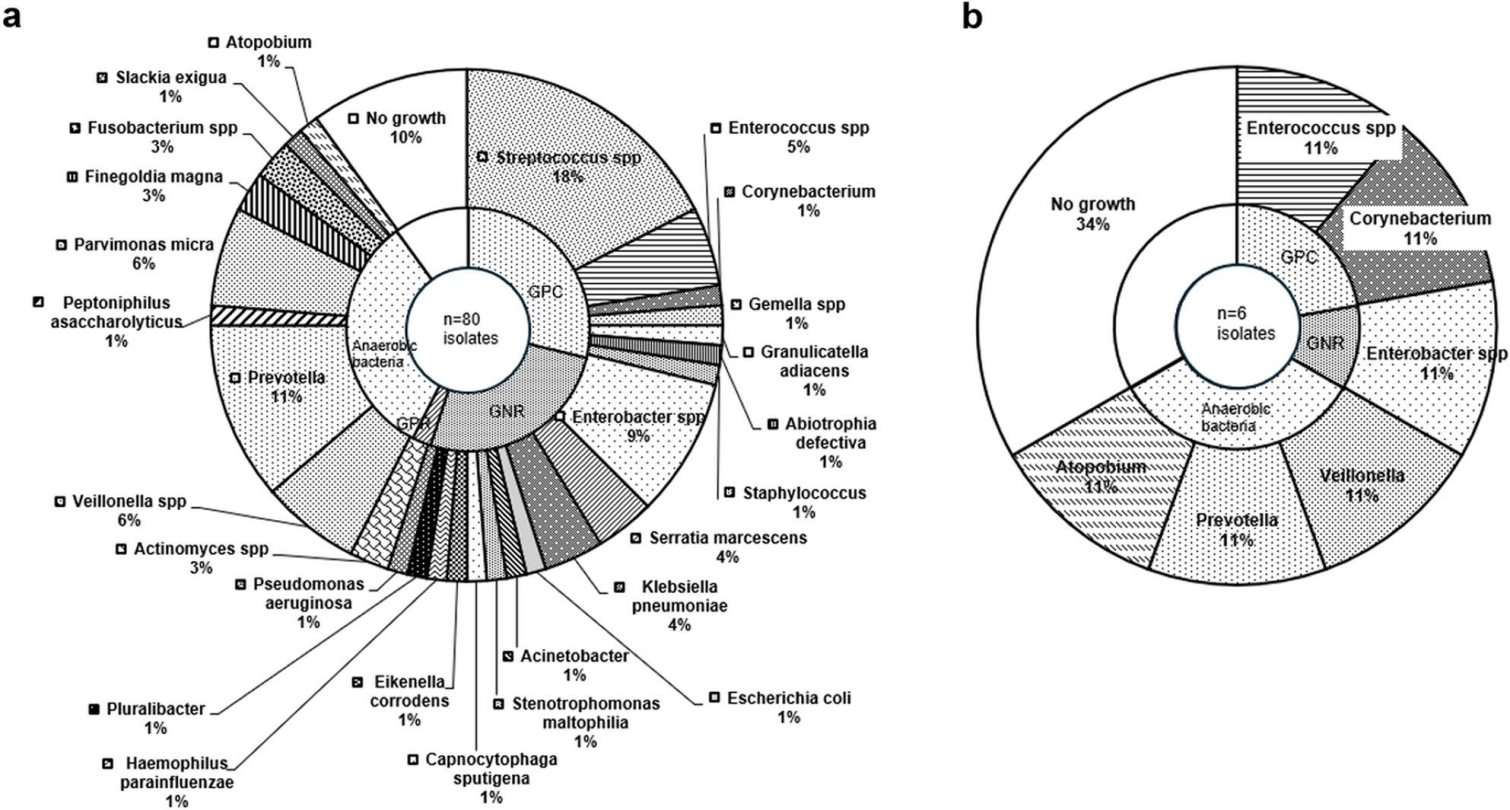
Distribution of causative microorganisms isolated from surgical site infections (SSIs). **a** Conventional sutures group (**b**) Triclosan-coated sutures group. The “n” represents the total number of isolated organisms (including multiple isolates per patient), not the number of patients. The triclosan group exhibited a lower detection rate of Gram-negative rods and a higher proportion of culture-negative results

The causative organisms identified in SSI are shown in Fig. 1. A wide variety of bacterial species was detected in both groups. Because multiple organisms could be isolated from a single patient, the total number of isolates exceeded the number of SSI cases. However, the triclosan group demonstrated a lower detection rate of Gram-negative rods and a higher proportion of culture-negative cases.

## Discussion

This study investigated the efficacy of triclosan-coated antimicrobial sutures for superficial wound closure and intra-abdominal use, including reinforcement of the staple line of the gastric conduit and anastomotic sites, in oesophageal cancer surgery. The incidence of superficial, deep, and organ/space SSIs showed a declining trend in the triclosan group, although statistical significance was only observed for the overall SSI rate (total SSI, 25.9% vs. 10.6%). These findings suggest that the expanded use of antimicrobial sutures may significantly reduce the overall risk of SSI in oesophageal cancer surgery.

Although the overall SSI rate was significantly lower in the triclosan group, the differences in superficial and deep SSIs did not reach statistical significance. This may be partly attributable to the limited number of events in each category, reducing the statistical power to detect differences. In addition, variability in case mix, comorbidities, and tumour characteristics (e.g., tumour location and stage) may have influenced the distribution of SSI subtypes. Given the relatively small sample size of this study, these factors could have diluted the observed effects when the SSI subtypes were analysed separately.

Furthermore, in this exploratory multivariable logistic regression analysis, suture type remained statistically associated with SSI. However, given the limited number of events relative to the number of covariates included in the model, this finding should be interpreted cautiously. Additionally, an altered microbiological profile was observed in the triclosan-coated sutures group, with a reduced detection rate of Gram-negative rods and a higher incidence of culture-negative infections.

Postoperative SSIs are multifactorial complications influenced by patient- and procedure-related factors [17]. Patient-related factors, including advanced age, comorbidities, diabetes mellitus, obesity, smoking history, and malnutrition, have consistently been associated with an elevated risk of SSIs [18, 19]. Although some of these factors may be addressed through perioperative management, many remain difficult to modify intraoperatively, highlighting the importance of intraoperative infection prevention strategies. In gastrointestinal surgery, the risk factors for SSIs include sex, BMI, diabetes mellitus, ASA-PS, emergency surgery, smoking history, hypoalbuminemia, wound classification, and prolonged operative time. In oesophageal cancer surgery, diabetes and higher ASA scores have been identified as potential risk factors for SSIs [4, 12]. Moreover, SSIs adversely affect postoperative outcomes, reinforcing the importance of effective prevention.

Multiple randomised controlled trials and meta-analyses have reported that triclosan-coated antimicrobial sutures reduce SSI rates when used for skin and fascial closure, with an estimated relative risk reduction of approximately 28% [5–8]. However, their efficacy may vary depending on the depth of infection. Although antimicrobial sutures are typically used for superficial closure, we evaluated their use in all suture applications, including anastomotic reinforcement and intra-abdominal sites, in oesophageal cancer surgery. The incidence of superficial, deep, and organ/space SSIs was reduced in the triclosan group, with a significantly decreased overall SSI rate (Table 2). Notably, the rate of anastomotic leakage decreased in the triclosan group, potentially reflecting improvements in surgical techniques or anastomotic skills over time, although the possibility that antimicrobial sutures contributed to a reduced bacterial burden surrounding the anastomotic site, thereby supporting local wound healing, cannot be excluded. One plausible mechanism is that triclosan-coated sutures used for reinforcement at the gastric conduit staple line or anastomotic site may inhibit bacterial colonisation on the suture material, thereby preventing localised infection and inflammation that could compromise tissue integrity, causing leakage. Nevertheless, given the historical nature of the comparison, the reduction in clinically significant (Clavien–Dindo grade ≥ 3) anastomotic leakage may be influenced by this methodological limitation. In microbiological cultures from infected sites, the detection rate of bacteria was lower in the triclosan group, suggesting suppression of bacterial colonisation (Fig. 1). Triclosan-susceptible organisms such as *Streptococcus* spp. and *Escherichia coli* were rarely detected in this group. In contrast, less susceptible species, including *Enterococcus* spp. and anaerobes, were identified in both groups. These findings imply that although triclosan-coated sutures may reduce certain infections, they are not universally effective, thus requiring continued surveillance of pathogenic trends and appropriate perioperative antibiotic selection. Furthermore, the detection rate of Gram-negative rods was lower, whereas the proportion of culture-negative cases was higher in the triclosan group compared with the conventional suture group, suggesting that triclosan-coated sutures may suppress certain susceptible bacterial species, potentially contributing to the overall reduction in SSI incidence. Nevertheless, triclosan-coated sutures should be considered as only one element in a multimodal SSI prevention strategy, and optimal surgical techniques, perioperative care, and institutional infection control remain critical strategies.

This study included patients with varying disease stages and clinical backgrounds, which inevitably introduced some degree of heterogeneity. However, this reflects real-world surgical practice at our institution and was intended to maximise the sample size to ensure sufficient statistical power for the primary endpoint. Moreover, all patients underwent surgery following a standardised SSI prevention bundle and perioperative management protocol, thereby minimising variability in infection prevention measures. The analysis was therefore performed on the entire cohort to preserve statistical robustness, while acknowledging this clinical heterogeneity as a study limitation.

This study has some limitations. First, the comparison between the triclosan-coated and conventional suture groups was not conducted over the same period; the former included cases from 2024, whereas the latter included cases from 2021 to 2023. Therefore, the possibility of time bias cannot be excluded. Improvements in surgical techniques, greater operator expertise, or changes in perioperative management over time may have influenced the outcomes, independent of suture type. Second, the number of patients in the triclosan-coated suture group was relatively small, which may have limited the statistical power to detect differences, particularly in subgroup analyses. Furthermore, the number of SSI events relative to the number of covariates included in the multivariable model was limited. Therefore, the possibility of model overfitting and unstable estimates cannot be excluded. Consequently, the results of the multivariable analysis should be interpreted cautiously. Third, the proportion of patients with ASA-PS grade 1 was significantly higher in the triclosan-coated group. Although ASA-PS classification was adjusted for in the multivariable analysis, this baseline imbalance may have contributed to the observed differences in SSI incidence. Residual confounding cannot be completely ruled out. Fourth, the inclusion of cervical and thoracic/abdominal oesophageal cancers may have introduced procedural heterogeneity. Although their distribution was comparable between groups, this clinical diversity should be considered when interpreting the results. Finally, the single-centre setting and retrospective observational design limit the generalisability of the findings. Further multicentre prospective studies or randomised controlled trials are warranted to validate the efficacy of triclosan-coated sutures in preventing SSIs during oesophageal cancer surgery.

## Conclusions

In conclusion, triclosan-coated antimicrobial sutures were associated with a reduced incidence of postoperative SSIs after oesophageal cancer surgery. Although these findings should be interpreted cautiously given the retrospective design and limited number of events, triclosan-coated sutures may represent a simple adjunct strategy within a comprehensive SSI prevention program. Further prospective multicentre studies are warranted to validate these findings. 

## Supplementary Information

Additional file 1. Components of the SSI prevention bundle (NICE guideline NG125).

Additional file 2. CDC definitions of surgical site infection.

## Data Availability

All data generated or analysed during this study are included in this article.

## Abbreviations

ASA: American society of anaesthesiologists
ASAPS: American society of anaesthesiologists physical status
BMI: Body mass index
CDC: Centres for disease control and prevention
MIS: Minimally invasive surgery
OR: Odds ratio
SSI: Surgical site infection
