# Supplementary material for: Expanded use of triclosan-coated sutures for surgical site infection prevention in oesophageal cancer surgery
Source: BMC Surg. 2026 Apr 20;26:383. doi: 10.1186/s12893-026-03723-4 (PMC13251054; doi:10.1186/s12893-026-03723-4)
Supplement: Supplementary file 1 — Additional file 1. Components of the SSI prevention bundle (NICE guideline NG125). [file 12893_2026_3723_MOESM1_ESM.docx]

**Additional file 1.** Components of the SSI Prevention Bundle (NICE NG125-Based).

**Preoperative Measures:**

- Preoperative shower or bath on the day before surgery.
- Avoidance of hair removal unless necessary; if necessary, it should be performed with electric clippers.
- Administration of prophylactic antibiotics 30–60 min before skin incision. For prolonged procedures, additional doses are to be administered every 3 h intraoperatively or when the estimated blood loss exceeds 1.5 L.

**Intraoperative Measures**

- Use of alcohol-based chlorhexidine for skin antisepsis.

**Maintenance of normothermia**

- Oxygen saturation >95%.
- Blood glucose levels < 200 mg/dL in patients with diabetes.

**Postoperative Measures:**

- Surgical dressings are left intact for 48 h, unless indicated otherwise.
- Standardised postoperative wound care education is provided to all patients.
