# Supplementary material for: Expanded use of triclosan-coated sutures for surgical site infection prevention in oesophageal cancer surgery
Source: BMC Surg. 2026 Apr 20;26:383. doi: 10.1186/s12893-026-03723-4 (PMC13251054; doi:10.1186/s12893-026-03723-4)
Supplement: Supplementary file 2 — Additional file 2. CDC definitions of surgical site infection. [file 12893_2026_3723_MOESM2_ESM.docx]

**Additional file 2.** CDC definitions of surgical site infection (SSI).

• Superficial incisional SSI: Infection occurring within 30 days postoperatively, involving only the skin or subcutaneous tissue, and meeting at least one of the following criteria:

- Purulent drainage from the incision.

- Organisms isolated from aseptically obtained specimens.

- Local signs of infection (pain, redness, swelling, and heat) with deliberate opening by the surgeon (unless negative culture results are obtained).

- Diagnosis of SSI by the attending physician.

• Deep incisional SSI: Infection involving the fascial and muscle layers occurring within 30 days (or 90 days if an implant is placed) and meeting at least one of the following conditions:

- Purulent drainage from the deep incision.

- Deep incision dehiscence or deliberate opening with signs of infection.

- Evidence of abscess or infection on imaging, histopathology, or direct examination.

- Diagnosis by the attending physician.

• Organ/space SSI: Infection involving any organ or space manipulated during surgery, occurring within 30 days (or 90 days if an implant is placed) and meeting at least one of the following criteria:

- Purulent drainage from a drain placed in an organ/space.

- Organisms isolated from fluid or tissue in an organ/space.

- Evidence of infection on imaging, histopathology, or reoperation.

- Diagnosis by the attending physician.
